# Intelligent Veterinary Disease Management Driven by Knowledge Graph for Conservation Breeding of Captive Forest Musk Deer

**DOI:** 10.3390/vetsci13060602

**Published:** 2026-06-21

**Authors:** Dequan Guo, Xin Fan, Zijie Lan, Chengli Zheng, Dapeng Zhang, Zhenyu Wang, Minyao Tan

**Affiliations:** 1 School of Automation, Chengdu University of Information Technology, Chengdu 610225, China; 2 Sichuan Institute of Musk Deer Breeding, Sichuan Institute for Drug Control, Chengdu 611845, China; 3 Sichuan Chuanrongda Technology Co., Ltd., Chengdu 610041, China

**Keywords:** forest musk deer, joint entity-relationship extraction, forest musk deer health management, knowledge graph

## Abstract

In artificial breeding of forest musk deer (Moschus berezovskii), common diseases such as abscess, enteritis, pneumonia, and parasitic infections exhibit persistently high morbidity rates. The early symptoms of certain diseases are often insidious and difficult to discern. Conventional manual inspection routines not only fail to achieve accurate diagnosis but also frequently disturb the animals, induce stress responses, and consequently delay optimal treatment windows. To address this practical challenge, this study employs an improved BRW-GPLinker joint entity-relationship extraction approach to perform integrated extraction and structural organization of disease entities, symptom manifestations, etiological associations, and preventive and therapeutic measures from farming literature and clinical records, thereby constructing a disease knowledge graph for forest musk deer. Through the introduction of a Boundary-Aware Module for refined entity boundary detection, a Relative Distance Bias Module to mitigate pairing errors in dense contexts, and a Weighted Sparse Multi-label Cross-Entropy loss function to enhance recall for infrequent relations, the proposed model achieves an F1 score of 0.887 on a self-constructed dataset and demonstrates favorable generalization capability on medical-domain datasets. By transforming fragmented clinical logs and manuals into structured medical associations, this knowledge graph facilitates rapid retrieval of forest musk deer disease information, thereby enhancing veterinary decision-making efficiency and assisting forest musk deer health management.

## Simple Summary

In the artificial breeding of forest musk deer (Moschus berezovskii), common diseases, namely abscess, enteritis, pneumonia, and parasitic infections, exhibit persistently high morbidity rates, with early symptoms often being insidious and difficult to discern. Conventional manual inspection routines not only fail to achieve accurate diagnosis but also frequently disturb forest musk deer, induce stress responses, and delay optimal treatment, thereby compromising forest musk deer health. To address this practical challenge, we construct a disease knowledge graph using an improved BRW-GPLinker joint extraction model, which demonstrates favorable generalization performance on medical-domain datasets. This model is capable of automatically extracting core medical concepts from unstructured forest musk deer disease texts and establishing semantic linkages among them. The disease knowledge graph generated by this technology provides a structured data foundation for veterinary clinical decision-making, thereby facilitating intelligent prevention and management of forest musk deer diseases.

## 1. Introduction

The Forest musk deer (Moschus berezovskii) [1], a Class I protected wildlife species in China, produces musk that has exceptionally high value as both a fragrance and an essential component in traditional Chinese medicine [2]. However, the rapid expansion of captive breeding operations has precipitated severe health crises. Mortality and morbidity in captive musk deer predominantly arise from suboptimal husbandry and management practices. The main symptoms currently present are abscesses, respiratory tract infections, parasitic diseases, stress-related disorders, and gastrointestinal diseases. These challenges substantially compromise survival and reproductive performance, restricting the development of the sustainable industry [3]. Among these diseases, abscesses caused by mixed bacterial infections occur throughout the year without an obvious age trend and can lead to systemic infection and hinder population growth in severe cases [4,5,6]. Of these, pneumonia exhibits high morbidity with a significant mortality risk, occurs throughout the year in age groups, and can progress to systemic infections that impede population recovery in severe cases [7,8]; poly-parasitism in juveniles commonly results in growth retardation and elevated death rates [9,10,11]; and stress responses frequently precipitate immunosuppression and secondary infections [12]. Collectively, these threats substantially impede population development and farming of forest musk deer, thus necessitating more effective disease knowledge management strategies.

Currently, knowledge regarding the prevention and treatment of forest musk deer diseases—including common conditions such as pneumonia, parasitic infections, and abscesses—remains dispersed across disparate unstructured sources. Traditional manual organization proves inefficient in establishing cross-document relationships [12,13,14], severely hindering early disease identification and timely intervention. This underscores the necessity of efficiently processing unstructured veterinary records and providing structured data support for clinical decision-making. Natural language processing technology has emerged as a critical pathway for advancing intelligent husbandry management [15]. This technology enables the automated extraction of core entities such as specific forest musk deer diseases, symptoms, and treatment regimens from textual sources, and accurately identifies clinical logical relationships among them (e.g., “manifests as” or “used to treat”), thereby constructing structured knowledge representations. Because forest musk deer disease knowledge remains highly fragmented, health management in artificial breeding settings suffers from inadequate information support, making structured knowledge integration essential. Joint entity and relation extraction for knowledge graph construction presents a viable approach to assisting disease diagnosis and reducing unnecessary human intervention, thereby assisting forest musk deer health management.

Joint extraction has emerged as the dominant paradigm for the construction of domain-specific knowledge graphs [16,17,18,19]. Contemporary approaches differ primarily in decoding strategy. Cascade architectures such as CasRel employ hierarchical binary tagging to extract head entities prior to conditional tail entity and relation annotation [20]. Token-pair-linking methods exemplified by TPLinker reformulate extraction as matrix-based linking operations for end-to-end single-stage processing [21]. Decomposition-based strategies, such as PRGC, partition the task into subtasks such as relation determination, entity extraction, and subject–object alignment [22]. More recent advancements include unified triplet classification through OneRel [23] and bidirectional extraction processes through BiRTE [24]. However, the effectiveness of these methods is substantially compromised when applied to forest musk deer clinical texts, which are characterized by intricate entity nesting, ambiguous span boundaries, and relation sparsity. This degradation in performance undermines the quality of constructed knowledge graphs and restricts their utility for forest musk deer health management.

Improving joint extraction performance in specialized domains requires accurate entity boundary detection and effective modeling of relative positional relationships. In entity boundary detection, multi-scale feature extraction has shown promise for capturing discriminate characteristics. Lou et al. [25] demonstrated that multi-scale convolution operations enhance dairy cattle disease entity identification, while Zhang et al. [26] advanced this approach through entity-aware visual prompt injection for Chinese agricultural disease terminology. Explicit boundary modeling offers a complementary strategy: Wang et al. [27] developed parallel frameworks for simultaneous boundary detection and category classification, and Guo et al. [28] employed boundary-aware large language models to address low-resource named entity recognition scenarios. For modeling of positional relationships, Zhu et al. [29] incorporated relative position encoding within cascade decoders for the extraction of biomedical relationships, Su et al. [30] introduced rotational position embedding to model relative dependencies via rotation matrices, and Raffel et al. [31] proposed relative positional bias mechanisms employing logarithmic binning and directional decay to capture local distance dependencies. Despite advances in both research lines, existing frameworks typically maintain modular boundary detection and positional modeling as separate components. This architectural separation, though effective in general domains, complicates joint optimization for forest musk deer disease texts that simultaneously exhibit entity nesting and relation sparsity, directly impacting the reliability of disease-assisted diagnosis in artificial breeding settings. When boundary detection and relational pairing operate independently, extraction errors propagate through downstream applications, leading to incomplete disease knowledge graphs. These graphs fail to capture critical associations between conditions such as abscess, enteritis, or pneumonia and their corresponding symptoms or treatments. Such gaps ultimately constrain early disease identification, increasing unnecessary manual inspection.

To address the challenges of joint entity and relation extraction, thereby supporting disease-assisted diagnosis, reducing human intervention, and improving disease prevention in the artificial breeding of forest musk deer, this study proposes BRW-GPLinker, an ensemble framework built on the Global Pointer architecture [32]. When processing unstructured disease texts for forest musk deer, one frequently encounters complex entity nesting and relation overlap phenomena. Traditional pipeline models or other joint extraction models often perform poorly when confronted with such intricate structures. The GlobalPointer architecture formulates entity recognition as a global scoring problem over token pairs, employing a globally oriented matrix design that scores all possible spans simultaneously through a unified scoring function. This matrix-based formulation is inherently well-suited to handling nested and overlapping entities, as it avoids the cascading decoding errors typical of sequence-labeling approaches and permits direct modeling of span interactions. Therefore, it was adopted as the baseline model, upon which targeted enhancement modules were introduced to further address problems such as boundary ambiguity. To tackle the issue of nested and ambiguous entity spans that hinder accurate identification of diseases such as pneumonia, parasitic infections, and abscess, we design a Boundary-Aware Module (BAM) that strengthens entity edge features through multi-scale convolutions and gating mechanisms, enabling precise span detection for improved early disease recognition. To address high-density local pairing errors prevalent in densely annotated veterinary texts, we propose a Relative Distance Bias Module (RDBM) that embeds distance-aware bias into the token-pair linking process, effectively rectifying erroneous associations and enhancing the reliability of disease knowledge graph construction. To overcome sparsity-induced omission caused by long-tail relation distributions, we introduce a Weighted Sparse Multi-label Cross-Entropy (WSMCE) loss that rebalances class weights, ensuring comprehensive coverage of associations between diseases and other entity nodes critical for knowledge graph-assisted forest musk deer disease management. Experimental results demonstrate that our proposed model achieves superior performance on joint extraction tasks compared to the baseline, validating its effectiveness and practical value in the domain of forest musk deer diseases. Furthermore, validation on the CMeIE-V2 medical dataset exhibits favorable generalization capability, providing a technical foundation for subsequent intelligent veterinary information management.

## 2. Materials and Methods

### 2.1. Materials

We employed a multi-source data collection strategy encompassing: (1) clinical records from standardized captive breeding facilities documenting disease presentation and therapeutic management; (2) authoritative veterinary manuals and professional monographs endorsed by regulatory institutions; and (3) systematically retrieved academic publications from established authoritative databases.

Clinical records constitute a significant data source, aggregated from daily health and disease management logs maintained at two standardized artificial breeding facilities housing approximately one thousand forest musk deer in total. These logs document observations of major diseases, typical symptoms, and corresponding treatment management measures. To ensure data reliability and representativeness, these empirical records were systematically integrated and cross-validated against authoritative husbandry manuals. This multi-source data strategy yields practically valuable insights and establishes a solid foundation for constructing a domain-specific dataset for forest musk deer diseases.

We consulted authoritative veterinary manuals and professional monographs published by recognized industry experts and regulatory institutions. Primary references included “Forest Musk Deer Breeding Technology and Musk Production (2025)” and “Artificial Breeding and Scientific Farming of Forest Musk Deer (2020)”. These complementary sources provide comprehensive, evidence-based wildlife farming veterinary protocols and breeding guidelines covering captive propagation, health management, behavioral enrichment, and sustainable musk extraction, all officially endorsed by the relevant authorities.

We systematically retrieved peer-reviewed publications from authoritative databases including China National Knowledge Infrastructure (CNKI), and Wanfang Data, spanning from 2000 to the present, and cross-validated sources to ensure reliability. Inclusion criteria were strictly limited to empirical studies explicitly addressing forest musk deer pathology, clinical characteristics, or intervention strategies; exclusion criteria eliminated non-peer-reviewed literature, duplicate publications, and articles lacking specific descriptions of medical entities and their relationships. This multi-source corpus primarily encompasses common forest musk deer diseases such as abscesses, enteritis, pneumonia, and parasitic infections, along with their etiology, clinical manifestations, and intervention strategies. The collected knowledge provides a reliable textual foundation for constructing disease knowledge graphs, further demonstrating the model’s validity and practical utility in the domain of forest musk deer diseases.

Given the severe fragmentation of existing text resources on forest musk deer diseases, we developed MS-Data, a specialized corpus tailored for joint entity-relation extraction tasks in this domain. Data collection followed a multi-source strategy designed to maximize authenticity, professional authority, and topical comprehensiveness, encompassing common conditions, disease symptoms, and drug information. Table 1 includes MS-Data definitions of entity types, relationship types, and examples of triples. To ensure data quality and usability in the forest musk deer disease domain, the raw data underwent rigorous filtering. Specifically, preprocessing eliminated noise through entry deduplication, terminology normalization, and expression standardization.

**Figure 1:**
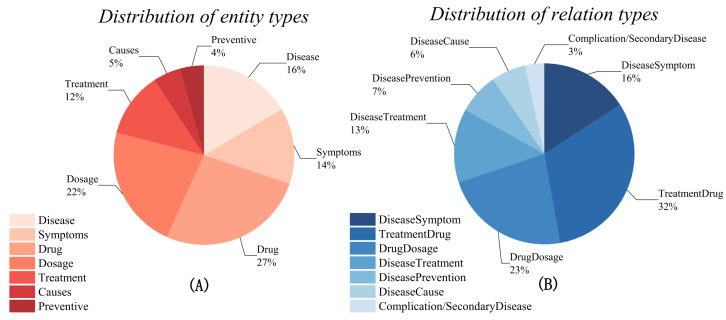
Statistical distribution of the MS-Data. Panel (**A**) shows the distribution of disease data for musk deer entity types, while panel (**B**) shows the distribution of disease data for musk deer relationship types.

The MS-Data covers seven entity types (e.g., disease, symptoms, drug) and seven relation types (e.g., disease–symptoms, drug–treatment), comprising 8310 labeled entities and 7074 annotated relational pairs. This includes common conditions such as abscess, enteritis, pneumonia, and parasitic infections. A detailed statistical analysis is presented in Figure 1, including entity type and relation type distribution, percentage, and data volume.

The MS-Data dataset was annotated by a single researcher following strictly predefined entity and relation labeling guidelines. To minimize errors and ensure high annotation quality, the researcher conducted multiple rounds of rigorous self-inspection. Following this self-review, two senior researchers with expertise in forest musk deer veterinary medicine independently reviewed all labeled entities and relations. Any ambiguous or inconsistent cases were jointly discussed and rectified until consensus was reached. This dual-review and consensus-driven process ensures high accuracy and reliability in the construction of the forest musk deer disease dataset. Following preprocessing, approximately 50,000 characters of structured text covering common diseases such as pneumonia, parasitic infections, and abscess were organized and partitioned into training and validation sets in an 8:2 ratio, comprising 903 and 212 valid records, respectively. This partition provides sufficient data for model training, hyperparameter tuning, and performance evaluation. Additionally, the CMeIE-V2 Dataset [33], a widely recognized benchmark for the extraction of Chinese medical information, was used as an auxiliary test to assess the cross-domain generalization capacity of the proposed model.

### 2.2. Model

#### 2.2.1. Overall Model

**Figure 2:**
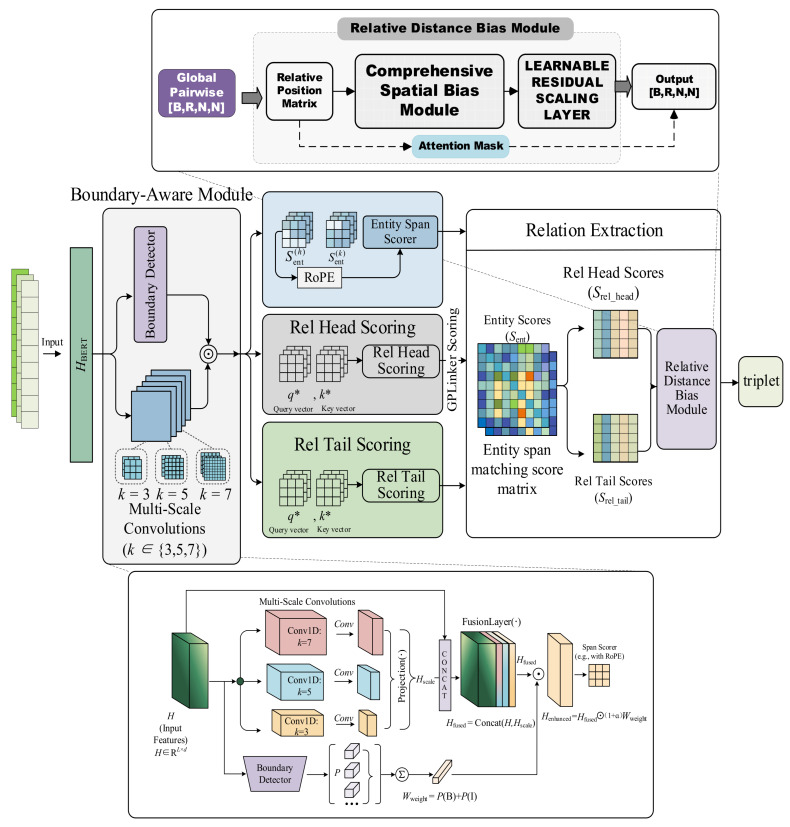
The BRW-GPLinker architecture. First, the BERT-encoded text enters the Boundary-Aware Module (BAM(gray)), where boundary detection and multi-scale convolution features are fused to yield boundary-sensitive representations. These are then fed in parallel to three branches: the Entity Span Scorer (blue) employs RoPE to evaluate candidate spans, while the Rel Head Scoring (gray) and Rel Tail Scoring (green) modules independently score boundary correspondences via query and key projections. Finally, the Relation Extraction block aligns entity scores with these streams to produce relation head and tail scores, which are refined by the Relative Distance Bias Module (purple)to generate the final triplets.

The general architecture of our proposed BRW-GPLinker model is presented in Figure 2. As an improved variant of GPLinker, this joint extraction model is specifically designed to enhance entity recognition and relation extraction performance in forest musk deer disease texts, ultimately supporting disease-assisted diagnosis and reducing unnecessary human intervention in artificial breeding settings. The model consists of four core components: a feature layer, an encoding layer, an extraction layer, and a loss function. In the encoding layer, the pre-trained BERT model [34] is used to extract contextual semantic representations from the input text sequence. In the feature layer, the Boundary-Aware Module effectively enhances entity edge features using multi-scale convolutions and a gated boundary detector, enabling precise localization of nested and ambiguous entity spans for improved identification of diseases such as pneumonia, parasitic infections, and abscess. Concurrently, the Relative Distance Bias Module in the extraction layer introduces learnable positional constraints into the global pointer scoring matrices, effectively correcting entity pairing errors in high-density regions and strengthening spatial position associations, thereby enhancing the reliability of disease knowledge graph construction. Finally, a Weighted Sparse Multi-label Cross-Entropy loss function is applied to alleviate the long-tail relation sparsity issue, ensuring comprehensive coverage of associations between diseases and other entity nodes critical for knowledge graph-assisted forest musk deer disease management, and outputting the globally optimal set of entity-relation triples for the given input.

#### 2.2.2. Boundary-Aware Module

**Figure 3:**
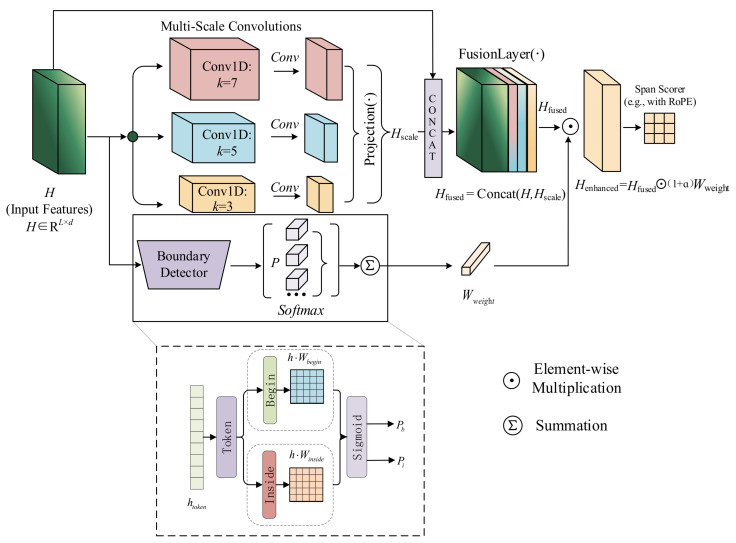
Architecture of the Boundary-Aware Module. Different colored feature maps represent semantic features extracted by convolution kernels with different receptive fields. The lower sub-module illustrates the generation of boundary-aware weights from BIO-based boundary information, which are used to adaptively enhance entity boundary representations before feature fusion.

To address the challenges of severely nested medical terminology and boundary ambiguity in forest musk deer disease texts, this study proposes the Boundary-Aware Module. Grounded in multi-scale feature extraction, this module enhances feature recognition of critical entity regions through an integrated gated boundary detection mechanism, thereby mitigating entity nesting and boundary ambiguity in forest musk deer disease texts. Specifically, following the acquisition of semantic representations with enhanced entity boundary features at the feature layer, the encoding layer employs multi-scale feature extraction and a dedicated boundary detector to project these features into start and end point vectors corresponding to each entity type, thereby providing a solid foundation for subsequent entity span scoring and relational triple computation. Figure 3 illustrates the detailed structure of the Boundary-Aware Module.

Although the BERT model excels at capturing long-range contextual semantics, it often exhibits insensitivity to local features when processing medical texts. Medical entities vary significantly in length; for instance, “fever” is a short word, whereas “purulent pneumonia” is a compound term. A single-sized convolution kernel struggles to capture these diverse features simultaneously. Therefore, this paper proposes a parallel multi-scale extraction module. Given a sentence $X\;=\;[X_{1},X_{2}{,\ldots X}_{n}]$ that contains tokens, we first employ the BERT encoder to extract context-aware high-dimensional latent representations for each token in X, yielding a representation matrix $H\;\in \;R^{n\times v}$, where v denotes the embedding dimension. This process establishes semantic mappings within a continuous vector space, providing the foundational basis for multi-scale feature reconstruction, which is represented as follows:

(1) $$h_{1},h_{2},\ldots h_{n}=\mathrm{BERT}(X_{1},X_{2},\ldots X_{n})$$

(2) $$H_{\mathrm{scale}}=\mathrm{Projection}\left(\underset{k\in 3,5,7}{⊕}\mathrm{ReLU}\left({\mathrm{Conv}}_{k}\left(H\right)\right)\right)$$

(3) $$H_{\mathrm{fused}}=\mathrm{FusionLayer}\left(H+H_{\mathrm{scale}}\right)$$

where k denotes the convolution kernel size. This paper employs k ∈ {3,5,7} this mechanism to construct a parallel multi-scale scanning architecture, where the Projection () module concatenates multi-scale convolutional features, and the Fusion Layer () fuses the original feature map H with $H_{\mathrm{scale}}$ to mitigate potential semantic information loss.

This paper presents a boundary detector based on gated attention. Its key innovation lies in using the sum of the predicted probabilities of the ‘B’ (beginning) and ‘I’ (inside) tags from the BIO tagging scheme as gating signals, which offers clearer physical interpretability than conventional weights. To deceptively enhance entity boundaries for musk deer disease entities of varying lengths, the detector incorporates parallel multi-scale convolutions. Specifically, the model first predicts BIO labels [35] for each token, then computes the gating weight as the combined probability of the start and inner positions of the entity. This design achieves a high signal-to-noise ratio, effectively highlighting entity regions while suppressing background noise. By strengthening entity feature discrimination, it notably improves recall for long-tailed and complex-boundary entities, which is represented as follows:

(4) $$L_{\mathrm{bound}}={\mathrm{MLP}}_{\mathrm{bound}}\left(H\right)$$

(5) $$W_{\mathrm{weight}}=\mathrm{Softmax}\left(L_{\mathrm{bound}}\right)\left[:,B\right]+\mathrm{Softmax}\left(L_{\mathrm{bound}}\right)\left[:,I\right]$$

where $L_{\mathrm{bound}}$ denotes the boundary prediction matrix, which is the matrix of predicted results output by the model. The ${\mathrm{MLP}}_{\mathrm{bound}}$ maps high-dimensional input features into the BIO three class classification space, while $\mathrm{SoftMax}(L_{\mathrm{bound}})[:B]$ and $\mathrm{SoftMax}(L_{\mathrm{bound}})[:I]$ map the entity head and tail vectors into probability distributions respectively.

The final enhanced features $H_{\mathrm{enhanced}}$ are obtained by element-wise multiplication of the fused features $H_{\mathrm{fused}}$ with the boundary weights, which are represented as follows:

(6) $$H_{\mathrm{enhanced}}=H_{\mathrm{fused}}⊙\left(1+\alpha \cdot W_{\mathrm{weight}}\right)$$

#### 2.2.3. Encoding Layer

The resulting sentence representation is obtained as follows, employing two feedforward layers, which are represented as follows:

(7) $$q_{i,a}=W_{q,a}h_{i}^{\prime }+b_{q,a}$$

(8) $$k_{i,a}=W_{k,a}h_{i}^{\prime }+b_{k,a}$$

The score for an entity of type *α* over the span $S_{a}(i:j)$ from position i is j calculated as follows, which is represented as follows:

(9) $$\begin{array}{ll} S_{a}(i:j) & ={\left(R_{i}q_{i,a}\right)}^{T}\left(R_{j}k_{j,a}\right)\\ & =q_{i,a}^{T}R_{j-i}k_{j,a} \end{array}$$

where ${R_{i}}^{T}R_{j}$ is used primarily to determine the position information of an entity or relation within the matrix, $R_{i}$ denotes the rotation matrix for position i and $R_{i}q_{i}$ injects position information i into the start vector and $R_{i}k_{i}$ injects position information j into the end vector.

#### 2.2.4. Relative Distance Bias Module

**Figure 4:**
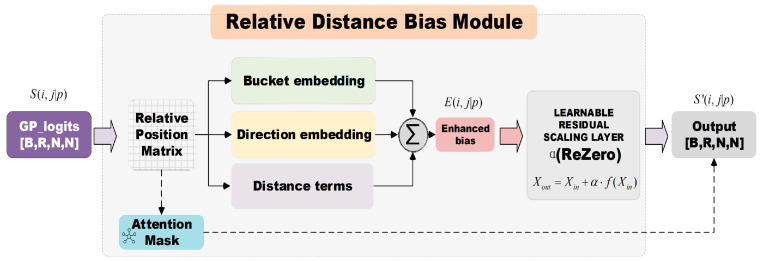
Structure of the relative distance bias module with adaptive fusion.

To address the pairing ambiguity arising from densely distributed identical entities in forest musk deer disease texts, this study proposes the Relative Distance Bias Module. Operating within the GPLinker framework, we adapt the relative position bias mechanism from T5 [31] and re-engineer it through two key enhancements: a relative distance term and an ReZero mechanism [36]. Specifically, inspired by the relative position bias of T5, this module injects a relation-specific distance bias into the scoring matrix to better capture disease–symptom and disease–treatment associations critical for early identification of conditions such as pneumonia, parasitic infections, and abscesses. Its design incorporates four key components: a logarithmic binning function for adaptive distance encoding, directional discrimination to distinguish semantic orientations between entity pairs, learnable per-relation parameters for flexible adaptation to diverse veterinary associations, and adaptive fusion via the ReZero mechanism to preserve base model capabilities while enhancing positional awareness. The detailed architecture of the Relative Distance Bias Module is illustrated in Figure 4.

GPLinker decomposes the extraction of the relationship into a combination of scores across five components, thereby transforming the extraction of the relationship into a scoring function for quintuples, which is represented as follows:

(10) $$S(s_{h},s_{t},p,o_{h},o_{t})=S(s_{h},s_{t})+S(o_{h},o_{t})+S(s_{h},o_{h}|p)+S(s_{t},o_{t}|p)$$

where $S_{h}$ and $S_{t}$ the head and tail indices of the subject, $O_{h}$ and $O_{t}$ denote the head and tail indices of the object, $S(s_{h},s_{t})$ denotes the start and end scores of the subject entity, $S(o_{h},o_{t})$ denotes the start and end scores of the object entity, and p denotes a predefined relation type.

The original GPLinker loss function has been modified, which is represented as follows:

(11) $$S(s_{h},s_{t},p,o_{h},o_{t})=S(s_{h},s_{t})+S(o_{h},o_{t})+S^{\prime }(s_{h},o_{h}|p)+S^{\prime }(s_{t},o_{t}|p)$$

where $S^{\prime }(s_{h},o_{h}\left|p\right)$ and $S^{\prime }(s_{t},o_{t}\left|p\right)$ denote the relation mapping score enhanced by the Relative Distance Bias Module, calculated as follows.

(12) $$S^{\prime }\left(i,j|p\right)=S\left(i,j|p\right)+\alpha \cdot E\left(i,j|p\right)$$

where α denotes the learnable scaling parameter of the ReZero module, and E(i,j|p) represents the distance bias term.

By introducing ReZero, the initialization scheme is introduced, where the residual weight is initialized to zero. This configuration ensures that the model initially degenerates into a standard GPLinker architecture during the early training phase, thus prioritizing the learning of robust semantic features before progressively incorporating positional biases through the learnable parameter α once semantic convergence is achieved.

Regarding the relative distance bias term E(i,j|p), it is formulated as a hybrid structure integrating discrete embedding components with continuous functional mappings, expressed as follows:

(13) $$E(i,j|p)=\underset{\mathrm{Bucket}}{\underbrace{{\mathrm{Emb}}_{\mathrm{bucket}}^{\left(r\right)}\left(f\left(d\right)\right)}}+\underset{\mathrm{Direction}}{\underbrace{{\mathrm{Emb}}_{\mathrm{dir}}^{\left(r\right)}\left(y\left(d\right)\right)}}+\underset{\mathrm{Distanceterms}}{\underbrace{E_{\mathrm{continuous}}(i,j|p)}}$$

where d=i−j denotes the relative distance between the positions of the subject and the object, ϕ(d) represents the logarithmic binning function and ψ(d) denotes the directional discrimination function. The logarithmic binning function is denoted as ϕ(d), which is represented as follows:

(14) $$\phi (d)=\mathrm{offset}(d)+\{\begin{array}{ll} \left|d\right|, & 0\leq \left|d\right|<M\\ {}[M+\frac{\log(\left|d\right|/M)}{\log(D_{\max}/M)}(K^{\prime }-M-1)], & M\leq \left|d\right|<D_{\max}\\ K^{\prime }-1, & \left|d\right|\geq D_{\max} \end{array}$$

where M denotes the guaranteed model considering neighboring entity pairs, $D_{max}$ represents the maximum perception distance, and entity pairs exceeding this distance are grouped into the same long-range bucket, $K^{\prime }$ denotes the total number of unilateral buckets; and offset(d) maps the forward and backward distances to non-overlapping numerical spaces.

The directional discrimination function is denoted as ψ(d), which is represented as follows:

(15) $$\psi (d)=\{\begin{array}{cc} 0, & d<0\;(\mathrm{Subject}\;\mathrm{follows}\;\mathrm{Object})\\ 1, & d=0\;(\mathrm{Same}\;\mathrm{position})\\ 2, & d>0\;(\mathrm{Subject}\;\mathrm{precedes}\;\mathrm{Object}) \end{array}$$

Following [37], to incorporate physical prior knowledge, the distance decay parameters are learned independently for each relation, which is represented as follows:

(16) $$E_{\mathrm{continuous}}(i,j|p)=\underset{\mathrm{Local}\;\mathrm{Prior}}{\underbrace{w_{L}^{\left(p\right)}\cdot \exp\left(-\frac{\left|d\right|}{\mathrm{softplus}(t^{\left(p\right)})|}\right)}}-\underset{\mathrm{Global}\;\mathrm{Penalty}}{\underbrace{w_{G}^{\left(p\right)}\cdot \frac{\log(1+\left|d\right|)}{\log(1+D_{\max})}}}$$

where $w_{L}^{\left(p\right)}$ and $w_{G}^{\left(p\right)}$ denote the learnable weights for local enhancement and global suppression, respectively, and $\tau^{\left(p\right)}$ controls the decay rate of the local correlation strength.

When processing variable-length sequences, the Transformer model pads short sentences to a uniform length. To prevent the bias term from being incorrectly applied to these invalid positions, an attention mask is employed to ensure computations are performed solely on valid token pairs, which is represented as follows:

(17) $${\mathrm{Output}}_{i,j}^{\left(p\right)}=\{\begin{array}{ll} S(i,j|p)+\alpha \cdot E(i,j|p), & \mathrm{if}\;{\mathrm{Mask}}_{i}=1\;\mathrm{and}\;{\mathrm{Mask}}_{j}=1\\ S(i,j|p), & \mathrm{otherwise} \end{array}$$

where Mask denotes the vector of the attention mask of the sentence.

#### 2.2.5. Weighted Sparse Multi-Label Cross-Entropy

To address sample imbalance and data sparsity in the forest musk deer disease dataset, we propose a Weighted Sparse Multi-label Cross-Entropy loss algorithm built upon GPLinker’s sparse multi-label cross-entropy framework. Combined with a fixed prior weighting strategy, this design suppresses training oscillation and enhances extraction of clinically significant long-tail relations. Following [38], the refined weighted sparse multi-label cross-entropy loss introduces asymmetric weighting factors to address class imbalance by imposing stronger penalties for false negatives, particularly in entity boundary classification where missed detections of conditions such as abscess, enteritis, or pneumonia could have serious consequences. To prevent training oscillations resulting from insufficient positive samples, a fixed prior weighting strategy is employed to ensure that the model consistently prioritizes long-tail positive samples throughout the training process, avoiding local optima and ensuring robust extraction of infrequent but clinically significant relations essential for reducing disease incidence. The formulation is represented as follows:

(18) $$L_{\mathrm{WSMCE}}(S,T)=w_{\mathrm{neg}}\log(1+\sum_{(i,j,t)\Omega_{\mathrm{neg}}}^{n}e^{S_{\mathrm{ij},t}})+w_{\mathrm{pos}}\log(1+\sum_{(i,j,t)\Omega_{\mathrm{pos}}}^{n}e^{{-S}_{\mathrm{ij},t}})$$

where s denotes the score matrix generating predicted score for all possible entity-relation triples; *T* represents the true label set; $\omega_{\mathrm{pos}}$ denotes the positive sample penalty weight, which amplifies the positive sample loss term; $\omega_{\mathrm{neg}}$ represents the tolerance level for incorrectly predicting a negative sample as positive; e is the natural constant; $\Omega_{\mathrm{pos}}$ denotes the set of positive samples with true labels; $\Omega_{\mathrm{neg}}$ denotes the set of negative samples with true labels.

The S scoring function is responsible for measuring entity boundaries and triplet relationships in the feature space, with its output serving directly as the input variable for the weighted sparse multi-label cross-entropy loss function $L_{WSMCE}$. The asymmetric weight coefficients introduced via the loss function enable closed-loop optimization of prediction evaluation and parameter updating, which is represented as follows:

(19) $$L_{\mathrm{Total}}=\frac{1}{3}(L_{\mathrm{Espan}}+L_{H}+L_{T})$$

where $L_{\mathrm{Total}}$ denotes the total loss for multi-task joint training; $L_{\mathrm{Espan}}$ represents the loss for entity span recognition; and $L_{H}$ and $L_{T}$ correspond to the discrimination losses for the start and end positions within a triplet, respectively.

Single-task loss, which is represented as follows:

(20) $$L_{X}=\frac{1}{\left|{\mathrm{Logit}}_{X}\right|}\sum_{i,j,\mathrm{type}}L_{\mathrm{WSMCE}}(\sigma \cdot ({\mathrm{Logit}}_{X}),T_{X})$$

where $\left|{\mathrm{Logit}}_{X}\right|$ denotes the score matrix size; $\sum_{i,j,type}L_{WSMCE}$ represents global index traversal; and ${\mathrm{Logit}}_{X}$ denotes the raw prediction matrix reflecting the model preliminary assessment of whether a specific head/tail entity pair will form a particular relation.

Entity span loss, which is represented as follows:

(21) $$L_{\mathrm{Espan}}=\frac{1}{\left|D_{\mathrm{Espan}}\right|}\sum_{a\in A_{\mathrm{entity}}}\sum_{i=1}^{n}\sum_{i=1}^{n}L_{\mathrm{WSMCE}}(s\cdot (s_{a}(i:j)),T_{a}(i,j))$$

where $T_{\alpha }\left(i,j\right)$ denotes the task ground truth label matrix; and $D_{\mathrm{Espan}}$ represents the normalization factor denoting the total number of possible entities span combinations; $A_{\mathrm{entity}}$ denotes the collection of predefined entity types; $\sum_{i=1}^{n}\sum_{i=1}^{n}L_{\mathrm{WSMCE}}$ performs a double traversal over all possible start positions and end positions in the sentence; and $s_{\alpha }\left(i:j\right)$ denotes the raw score generated by the entity scoring function.

### 2.3. Experimental Setup

#### 2.3.1. Parameters

We utilized PyTorch to implement our proposed BRW-GPLinker model on an RTX4090D GPU. The versions of Python and PyTorch were 3.12.1 and 2.5.1, respectively. The maximum number of iterations was set to 100. The parameters of our model are illustrated in Table 2.

For the MS-Data dataset, we configured a batch size of 8 and trained for 100 epochs, whereas for the larger-scale CMeIE-V2 dataset, we increased the batch size to 16 and reduced the training epochs to 50. In particular, the core architectural parameters remained consistent across both datasets: BERT dimension of 768 and hidden layer size of 64. This design strategy allows the model to adapt to varying dataset scales while maintaining architectural uniformity for fair comparison.

The results were evaluated using the F1 score metric. The F1 score is calculated based on the global True Positive (TP), False Negative (FN), and False Positive (FP) rates, reflecting the model classification performance across the entire set of relation triples, which is represented as follows:

(22) $$\mathrm{Precision}=\frac{\mathrm{TP}}{\mathrm{TP}+\mathrm{FP}}$$

(23) $$\mathrm{Recall}=\frac{\mathrm{TP}}{\mathrm{TP}+\mathrm{FN}}$$

(24) $$\mathrm{F1}\;\mathrm{score}=\frac{2\times \mathrm{Precision}\times \mathrm{Recall}}{\mathrm{Precision}+\mathrm{Recall}}$$

#### 2.3.2. Models

To comprehensively evaluate the effectiveness of the proposed model, we perform comparative experiments against seven representative joint-relation extraction methods, including CasRel [20], TPLinker [21], PRGC [22], PURE [39], OneRel [23], BiBRT [24], and GPLinker [32]. All competing models are implemented with the BERT-base Chinese pre-training language model as the backbone encoder to ensure a fair comparison. In addition, we perform ablation studies to assess the individual contributions of each key component in our model. Specifically, we systematically remove or add the Boundary-Aware Module, the Relative Distance Bias Module, and the Weighted Sparse Multi-label Cross-Entropy function to construct several model variants. These variants are evaluated under identical experimental conditions to isolate the effect of each proposed enhancement.

## 3. Results

### 3.1. Recognition Performances of Different Models

On the MS-Data benchmark, the proposed BRW-GPLinker achieves an F1 score of 0.887, representing a 2.0 percentage point improvement over GPLinker, and demonstrates favorable performance relative to CasRel, OneRel, and BiBERT. As shown in Table 3, this performance indicates that the integration of BAM and RDBM improves the location of the entity boundary and reduces the ambiguity of the pairing in nested scenarios. Furthermore, the WSMCE loss function effectively addresses the sparse relation distribution inherent in the forest musk deer disease domain. These results suggest that the proposed components contribute to a more reliable extraction performance.

Although the performance of all models on the CMeIE-V2 dataset is generally lower than that on the MS-Data dataset, which can be attributed to the distributional discrepancies between general medical corpora and domain-specific collections, the proposed BRW-GPLinker model achieves an F1 score of 0.590. Compared to GPLinker and other baseline methods, this model shows consistent improvements in both the F1 score and precision. These results suggest that the BAM, RDBM, and WSMCE strategies possess certain generalization capabilities. This indicates that the proposed enhancements are not limited to the extraction of forest musk deer disease, but also demonstrate potential utility in broader medical text processing scenarios.

To mitigate the impact of random initialization, all experiments were conducted across five distinct random seeds. The baseline GPLinker achieved an average F1 score of 0.864 ± 0.003, whereas the proposed model attained 0.883 ± 0.006. As detailed in Table 4, a paired t-test on the F1 scores confirmed that this improvement is statistically significant (*p* < 0.01).

### 3.2. Ablation Study

Comprehensive ablation experiments were conducted on the GPLinker baseline to systematically evaluate the contribution of each proposed component. As detailed in Table 5, eight model variants were constructed by selectively incorporating BAM, RDBM, and WSMCE. Where the checkmark (√) indicates that the corresponding module s(BAM, WSMCE, or RDBM) is integrated into the model variant.

Individual component analysis reveals distinct functional improvements. BAM independently improved F1 by 0.8% and recall by 1.6%, primarily improving the precision of the entity boundary detection. RDBM contributed a 0.7% improvement in F1 and a 0.4% gain in precision, optimizing relative positional modeling between paired entities. WSMCE alone increased both F1 and recall by 1.0%, effectively mitigating class imbalance through asymmetric loss weighting.

The combinations of module pairs yielded F1 improvements ranging from 1.1% to 1.2%, demonstrating additive effects between components. The complete integration of BRW-GPLinker achieved a cumulative gain in F1 of 2.0% with balanced improvements in both precision and recall.

These results demonstrate that the three modules exhibit complementary synergies in feature extraction, positional modeling, and loss optimization, forming an effective closed-loop enhancement mechanism.

### 3.3. Triple Extraction Results by Relation Category

**Figure 5:**
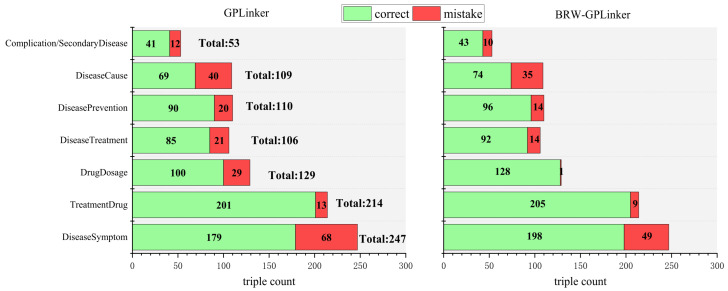
Comparison of correct and incorrect triples by relation category.

BRW-GPLinker achieves consistent performance gains across all seven relation categories in forest musk deer disease texts compared to the baseline GPLinker model. As shown in Figure 5, our model extracts more correct entity-relation triples while simultaneously reducing errors in every category.

The model demonstrates particular strength in critical relation categories. For Drug Dosage, correct triples increased from approximately 100 to 128 with erroneous predictions decreasing from 29 to 1. The DiseaseSymptom relation improved from 179 to 198 correct triples, with errors decreasing from 68 to 49. Disease Treatment and Prevention also demonstrated significant enhancement, with error reduction exceeding one third.

These results indicate that the proposed modules collectively mitigate the challenges of nested entities, ambiguous boundaries, and relation sparsity in domain-specific veterinary texts within wildlife farming. The reduction in errors in critical categories, such as DrugDosage, demonstrates the clear practical value of BRW-GPLinker for forest musk deer farming veterinary management applications.

### 3.4. Performance on Different Triples by Relation Category

**Figure 6:**
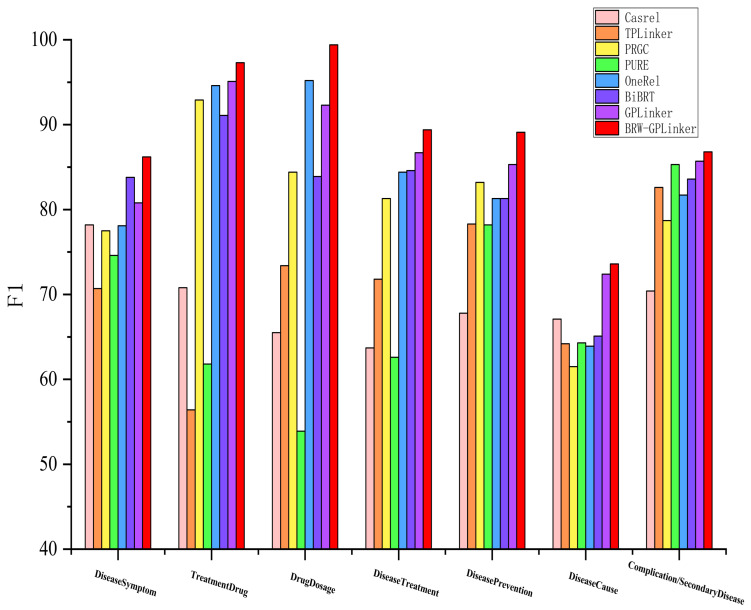
F1 score of extraction models on forest musk deer disease relations.

We evaluated BRW-GPLinker against seven baseline models in the seven relation categories in the forest musk deer disease knowledge graph. As shown in Figure 6, BRW-GPLinker achieves the highest F1 score in all categories. The model demonstrates particular strength in extracting medication-related entities, attaining F1 scores of 99.4% for ‘DrugDosage’ and 97.3% for ‘TreatmentDrug,’ along with 86.2% for DiseaseSymptom.

Compared to the highest performance baseline in each specific category, BRW-GPLinker shows consistent and objective performance gains. For DrugDosage, it improves the strongest baseline, OneRel, by 4.2 percentage points. For TreatmentDrug, it advances beyond GPLinker by 2.2 percentage points. In the DiseaseSymptom category, it exceeds BiBRT by 2.4 percentage points, suggesting that boundary-aware feature extraction effectively handles the complex entity nesting often found in symptom descriptions.

Consistent improvements are also observed across the remaining categories, where GPLinker serves as the strongest baseline. BRW-GPLinker surpasses it by 3.8 percentage points in DiseasePrevention, 2.7 percentage points in DiseaseTreatment, and 1.1 percentage points in Complication/Secondary. In particular, while DiseaseCause exhibits the lowest overall score across all models due to the inherent complexity of extracting causal relationships from veterinary texts, BRW-GPLinker still achieves a 1.2 percentage point improvement over GPLinker.

Overall, these results indicate that the integration of BAM, RDBM, and WSMCE contributes to improved performance across various relation types, demonstrating the effectiveness of BRW-GPLinker in joint entity and relation extraction tasks. Meanwhile, the resulting knowledge graph offers a structured foundation for organizing forest musk deer disease information.

### 3.5. Qualitative Analysis

**Figure 7:**
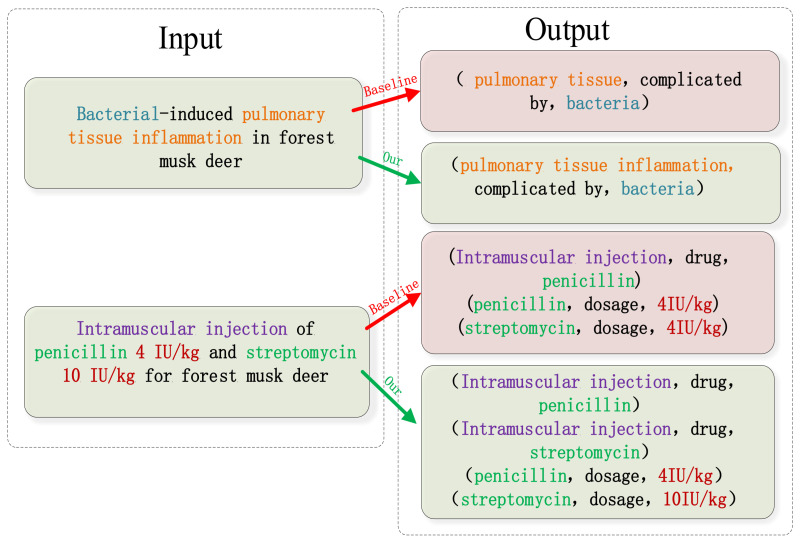
Case study comparison between GPLinker and BRW-GPLinker. Red and green arrows represent baseline and our model extractions, respectively; colored text highlights different entities and relations.

Qualitative comparisons between the baseline GPLinker (red arrows) and the proposed BRW-GPLinker (green arrows) in representative forest musk deer clinical texts are illustrated in Figure 7. In the bacterial pulmonary inflammation case, the baseline fails to recognize the core entity “inflammation,” resulting in incomplete triple extraction. In contrast, BRW-GPLinker successfully identifies the complete nested entity “pulmonary tissue inflammation” and correctly extracts the semantically consistent relation. In the medication regimen case, the baseline completely misses the streptomycin administration relationship and misclassifies dosage information, while BRW-GPLinker accurately captures all valid triples with precise relational structures.

These results demonstrate that the proposed model effectively remediates critical extraction failures of the baseline approach, including entity boundary detection errors, relation omission, and attribute misclassification. The resulting improvement in structured knowledge extraction accuracy provides a technical foundation for constructing more reliable forest musk deer disease knowledge graphs.

## 4. Discussion

### 4.1. Model Analysis

The results of BRW-GPLinker demonstrate its effectiveness, with its architecture achieving deep alignment with the linguistic characteristics of forest musk deer disease texts. Conventional baseline models often underperform when confronted with the dual constraints of entity nesting and relation sparsity, whereas the proposed architecture effectively circumvents parsing confusion arising from unstructured clinical texts. The synergistic interaction between the BAM and RDBMs successfully transforms structurally complex, highly ambiguous long sentences typical of forest musk deer disease descriptions into logically coherent two-dimensional scoring matrices of entity relations. Such sentences are characterized by dense, overlapping entities that readily induce pairing ambiguity. This ensures that analysis of high-density, overlapping symptoms is grounded in rigorous positional features and boundary rules.

The structural advantages of this architecture are particularly evident in critical and challenging relation categories. For instance, extraction of the “Disease-Symptom” relation frequently fails in conventional models due to highly nested terminological naming conventions. As observed in qualitative analysis, generic baseline models tend to rigidly fragment compound clinical terms such as “pulmonary tissue inflammation”; by contrast, the BAM provides robust boundary-aware capability, successfully preserving the semantic integrity of complete nested entities. Furthermore, for sparse relations such as “Drug-Dosage,” the WSMCE and RDBM play a critical calibration role. Rather than blindly conforming to high-frequency background noise, the model makes decisions more effectively based on distributional relationships and relative positions. This effectively minimizes extraction errors for critical dosage information, ensuring that the generated entity associations achieve not only higher quantitative metrics, but also demonstrate robust reliability in real extraction scenarios.

However, extraction performance for certain highly complex or infrequent relation types still indicates room for improvement. Future work will focus on optimizing the extraction of these challenging relations to further strengthen its effectiveness in veterinary information management and decision support. Overall, BRW-GPLinker is well suited to the forest musk deer disease domain, and results exhibit promising transferability to broader medical text mining tasks.

### 4.2. Partial Construction of the Forest Musk Deer Disease Knowledge Graph

**Figure 8:**
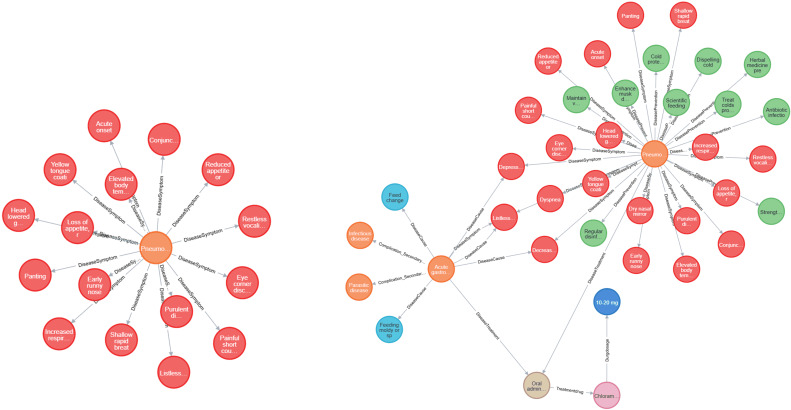
Partial knowledge graph of forest musk deer diseases. Different node colors represent specific entity categories: orange for Disease, red for Symptom, green for Prevention, blue for Cause, dark blue for Dosage, pink for Drug, and beige for Treatment.

Using the triples extracted by BRW-GPLinker from the MS-Data corpus, we constructed a partial knowledge graph for forest musk deer diseases, as shown in Figure 8. The graph schema comprises seven entity types—Cause, Disease, Dosage, Drug, Prevention, Symptom, and Treatment—and seven predefined relation categories, including Disease Symptom, Disease Treatment, Treatment Drug, Drug dosage, Disease Cause, Disease Prevention, and Complication/Secondary Disease. In total, the current graph contains 1359 entity nodes and 3386 relation triples, capturing core clinical knowledge such as symptom-disease associations (e.g., “rapid shallow breathing” linked to “pneumonia”), treatment regimens and drug-dosage chains. This structured representation enables preliminary intelligent querying for forest musk deer disease information. However, due to the inherent challenges of nested entities and sparse relations, the graph remains incomplete, particularly for low-frequency relation types such as DrugDosage and DiseaseCause. However, this partial graph serves as a technical foundation for further expansion, and future efforts will focus on incorporating external forest musk deer disease knowledge bases and annotating additional samples to improve coverage and extraction performance.

For the generated knowledge graph, we conducted domain expert validation to assess its clinical accuracy and practical utility. A random sample of 200 relation triples was extracted from the knowledge graph and independently reviewed by veterinary experts. The evaluation criteria focused on whether the extracted triples accurately reflected established forest musk deer disease entity relationships and whether the associations were correct and valid. The manual validation achieved an accuracy of 90%, confirming that the majority of constructed knowledge graph nodes are reliable. The primary issues among the erroneous entity relations were highly complex nested entities or excessively long entities.

Regarding practical utility, portions of the knowledge graph were utilized as a structured foundation for decision support. Qualitative analysis of selected subgraphs reveals that the left panel in Figure 8 presents a topology centered on “pneumonia.” This subgraph captures the disease and its associated entities, including specific symptoms such as rapid breathing and appetite loss, and directly links them to the disease node, while the right panel maps corresponding preventive measures and treatment regimens. This high degree of alignment with standard veterinary diagnostic logic demonstrates that the graph can facilitate symptom-based querying and disease management in artificial breeding environments.

### 4.3. Computational Cost Analysis

To evaluate the practical performance of the proposed architecture, we conducted a comprehensive computational cost analysis, comparing the baseline GPLinker with our progressively enhanced configurations from the perspectives of training time, Inference Time, Total Parameters, and GPU Memory consumption. As shown in Table 5, all evaluations were performed on a single NVIDIA GeForce RTX 4090 D GPU (24 GB VRAM) with a batch size of 8 and BERT-Base-Chinese.

According to the performance metrics presented in Table 6, the BRW-GPLinker architecture exhibits moderate computational overhead. Total parameters increased from 103.84 M to 108.96 M, with the increment primarily attributable to the BAM. Training time for 100 epochs increased from 40.2 min to 43.4 min. Single-sample inference time increased from 16.14 ms to 20.37 ms. GPU memory consumption peaked at 3.49 GB compared to 3.23 GB for the baseline. These increases fall within acceptable limits for deployment in forest musk deer farming environments.

These increases in parameters, training time, inference latency, and memory footprint are quantitatively modest relative to the baseline. The peak GPU memory consumption of 3.49 GB reflects modest hardware requirements, making it compatible with the limited computational resources of forest musk deer breeding facilities. The inference latency of approximately 20 ms per sample enables batch processing of clinical records without substantial throughput degradation. Given the constrained computational resources typical of specialized farming facilities, these characteristics indicate that the BRW-GPLinker architecture is technically feasible for deployment in forest musk deer breeding environments.

### 4.4. Limitations of Dataset Scale and Category Coverage

Although the proposed architecture demonstrates promising effectiveness in constructing forest musk deer disease knowledge graphs, the MS-Data dataset, comprising merely 903 training samples, remains within the small-sample regime for deep learning. This scale limitation introduces potential overfitting risks, wherein the model may excessively memorize formats, terminological conventions, or institution-specific annotation preferences from particular clinical records, thereby undermining its robustness when confronted with heterogeneous text sources or divergent clinical standards. Furthermore, constrained by the limited availability and partial scarcity of forest musk deer disease data, the current dataset primarily covers typical disease descriptions and has yet to fully encompass high-complexity scenarios such as comorbidity diagnosis, prolonged complex cases, or implicit clinical reasoning. This incompleteness, to some extent, restricts the model’s direct generalization capability across diversified clinical contexts.

To address the aforementioned limitations, future research will prioritize in-depth exploration along two dimensions. First, we will introduce transfer learning and semi-supervised learning frameworks to effectively reduce reliance on manual annotation by leveraging massive unlabeled veterinary clinical texts and cross-species disease datasets, thereby expanding the sample space and alleviating data sparsity. Second, we will explore large language model (LLM)-based few-shot learning mechanisms, aiming to rapidly adapt to emerging disease types and the dynamic evolution of clinical terminology through prompt engineering and domain-specific fine-tuning. By integrating these technical pathways, we anticipate further enhancing the model’s robustness and generalization capability in handling complex, diversified clinical data within practical forest musk deer conservation scenarios.

### 4.5. Practical Implications for the Forest Musk Deer Industry

Regarding the practical significance of this research for the forest musk deer industry, the proposed BRW-GPLinker model provides the core technical foundation for intelligent disease management. By efficiently extracting complex associations among diseases, typical symptoms, and treatment regimens, this model transforms massive, fragmented unstructured clinical logs and professional literature into a structured Forest musk deer disease knowledge graph. In practical disease prevention and clinical auxiliary diagnosis scenarios, this graph-based knowledge extraction demonstrates significant deployment potential. It can serve as the underlying infrastructure for intelligent information retrieval systems, enabling frontline veterinarians to access historical analogous cases and standardized intervention measures based on observed symptoms.

**Figure 9:**
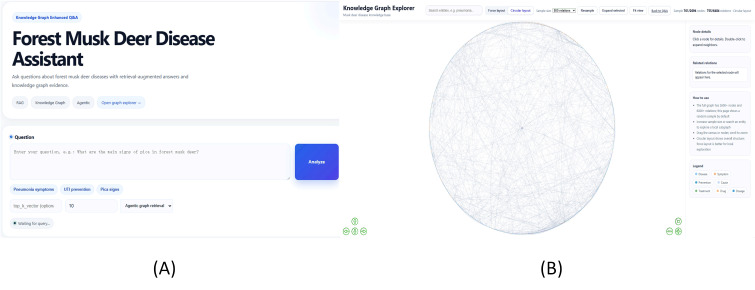
Prototype system of the Forest Musk Deer Disease Assistant. The platform integrates a RAG-based question-answering framework (**A**) with an interactive circular knowledge graph visualizer (**B**).

Moreover, the graph extraction framework established in this study offers certain expansion potential for subsequent intelligent forest musk deer farming technologies. While traditional structured knowledge graphs offer precision, they present high interaction barriers; integrating them with large language models can effectively close the implementation loop for practical deployment. To empirically demonstrate this utility, we developed a prototype platform named the Forest Musk Deer Disease Assistant, illustrated in Figure 9, which seamlessly bridges Retrieval-Augmented Generation (RAG)-driven querying with an interactive knowledge graph explorer. In future real-world applications within the forest musk deer disease domain, this knowledge graph can serve as an underlying domain-specific knowledge engine for large models, supporting complex pathological reasoning and multi-turn dialogue. This not only helps overcome the isolated state of knowledge graphs but also provides core data and logical support for constructing low-barrier, high-reliability professional veterinary auxiliary consultation systems tailored to forest musk deer health management.

## 5. Conclusions

This paper presents BRW-GPLinker, an enhanced joint entity-relation extraction model optimized for the forest musk deer disease domain. The architecture provides a robust foundation for systematic knowledge graph construction to support intelligent veterinary management. The model incorporates a Boundary-Aware Module to address ambiguous entity boundaries and nested structures commonly encountered in veterinary clinical texts, employs a relative distance bias mechanism to capture relational dependencies while reducing pairing errors, and utilizes an asymmetric weighted loss function to handle sparse relation distributions in wildlife farming contexts. Experimental results demonstrate that the proposed model achieves an F1 score of 0.887 with precision and recall of 0.893 and 0.881, respectively, on the self-constructed MS-Data dataset, outperforming baseline GPLinker and other joint extraction models. The model also exhibits moderate generalization ability on the general medical CMeIE-V2 benchmark, indicating its suitability for forest musk deer disease corpus processing. However, several limitations remain to be addressed in future work: (1) Pre-trained language models show advantages in medical knowledge-intensive tasks; therefore, integrating domain-specific large language models will be explored to further enhance entity boundary detection and relation reasoning for veterinary management applications. (2) Beyond the common challenges addressed herein, complex cross-sentence relations and implicit causal associations exist in forest musk deer disease corpora, which warrant further investigation to strengthen support for veterinary management practices. (3) The model’s computational efficiency when processing lengthy clinical documents still has room for improvement; more lightweight architectures will be explored to reduce inference burden while maintaining extraction accuracy, thereby better serving practical veterinary management needs in artificial breeding operations. (4) The current forest musk deer disease dataset remains limited in scale; future work will focus on expanding annotated samples and incorporating external veterinary sources to improve coverage of infrequent disease manifestations and relation types.

## Figures and Tables

**Table 1:** Definitions of entity types and relation categories in the MD-Data.

Entity Type	Definition	Relation Type	Definition of Relation	Example (Triplet)
Disease	Pathological conditions of forest musk deer.	DiseaseCause	The pathological or environmental origin of a disease.	(Gastroenteritis, DiseaseCause, Bacteria)
Symptoms	Clinical signs of a specific disease.	DiseaseSymptom	The correlation between a disease and its clinical manifestations.	(Pneumonia, DiseaseSymptom, Dyspnea)
Drug	Agents used for clinical intervention prescribed for a condition.	TreatmentDrug	Medication prescribed for a condition.	(Abscess, TreatmentDrug, Penicillin)
Dosage	Amount or frequency of medication for a drug.	DrugDosage	Administration details for a drug.	(Amoxicillin, DrugDosage, 20 mg/kg)
Treatment	Therapeutic procedures or strategies approach to treat a disease.	DiseaseTreatment	Strategies for prophylaxis and health management.	(Fracture, DiseaseTreatment, Splinting)
Prevention	Actions taken to inhibit disease spread.	DiseasePrevention	Strategies for prophylaxis and health management.	(Parasitism, DiseasePrevention, Deworming)
Causes	Clinical causes of disease.	Complication/ SecondaryDisease	Secondary conditions arising from a primary disease.	(Pneumonia, Complication/Secondary Disease, Laryngitis)

**Table 2:** Parameters of our proposed BRW-GPLinker model.

MS-Data		CMeIE-V2	
Parameters	Value	Parameters	Value
Batch size	8	Batch size	16
Learning rate	2 × 10^−5^	Learning rate	2 × 10^−5^
Bert dim	768	Bert dim	768
epochs	100	epochs	50
hidden_size	64	hidden_size	64
Optimization algorithm	Adam	Optimization algorithm	Adam

**Table 3:** Comparative experiments results on MS-Data and CMeIE-V2 datasets.

Model	MS-Data			CMeIE-V2		
	F1	Re	P	F1	Re	P
CasRel	0.702	0.654	0.764	0.482	0.477	0.488
TPLinker	0.781	0.776	0.798	0.502	0.498	0.507
PRGC	0.836	0.846	0.826	0.579	0.572	0.586
PURE	0.743	0.753	0.724	0.532	0.548	0.527
Onerel	0.842	0.808	0.879	0.585	0.586	0.583
BiBRT	0.844	0.824	0.864	0.539	0.504	0.579
GPlinker	0.867	0.833	0.903	0.583	0.525	0.630
Our	0.887	0.861	0.918	0.590	0.551	0.633

**Table 4:** Performance stability analysis under different random seeds.

Model	Mean *F*1	Std
GPLinker	0.864	0.003
BRW-GPLinker	0.883	0.006

**Table 5:** Ablation study of different modules in the BRW-GPLinker model.

Version	BAM	WSMCE	RDBM	F1	Re	P
GPLinker				0.867	0.833	0.903
Version1	√			0.875	0.849	0.898
Version2		√		0.877	0.843	0.904
Version3			√	0.876	0.838	0.907
Version4		√	√	0.878	0.855	0.905
Version5	√		√	0.878	0.823	0.914
Version6	√	√		0.881	0.854	0.910
BRW-GPLinker	√	√	√	0.887	0.861	0.916

**Table 6:** Computational cost comparison of different model configurations.

Model	Total Parameters	Training Time (100 Epochs)	Inference Time	GPU Memory
GPLinker	103.84 M	40.2 min	16.14 ms	3.23 GB
GPLinker + BAM	107.68 M	42.6 min	18.77 ms	3.42 GB
GPLinker + BAM + RDBM	108.96 M	43.3 min	20.31 ms	3.49 GB
BRW-GPLinker	108.96 M	43.4 min	20.37 ms	3.49 GB

## Data Availability

The original contributions presented in this study are included in the article. Further inquiries can be directed to the corresponding author.
